# Optimizing the route for production of activated carbon from *Casuarina equisetifolia* fruit waste

**DOI:** 10.1098/rsos.171578

**Published:** 2018-07-11

**Authors:** P. Ravichandran, P. Sugumaran, S. Seshadri, Altaf H. Basta

**Affiliations:** 1 Shri AMM Murugappa Chettiar Research Centre (MCRC), Taramani, Chennai 600 113, Tamil Nadu, India; 2 National Research Centre, Cellulose and Paper Department, El-Bohousse Street, Dokki 12622, Cairo, Egypt

**Keywords:** *Casuarina equisetifolia* fruit waste, alkali and acid activators, route of activation high-performance activated carbon, physico-chemical characteristics, adsorption capacities

## Abstract

This work deals with optimizing the conditions of pyrolysis and type of activator to upgrade the use of *Casuarina equisetifolia* fruit waste (CFW) as available and a potential precursor, in production of activated carbon (AC). In this respect, the route of activation was carried out through one- and two-step pyrolysis processes, using different chemical activating agents, such as H_3_PO_4_, KOH and ZnCl_2_. The performance of the CFW-based ACs is assessed by estimating the physico-chemical characteristics (pH, electrical conductivity, bulk density and hardness), surface morphology and scanning electron microscopy, together with carbon yield, surface area and adsorption performance of pollutants in aqueous medium (methylene blue, iodine and molasses colour removal efficiencies). The results show that the two-step activation process was more effective than one-step activation for providing high adsorption performance CFW-based ACs. The maximum Brunauer–Emmett–Teller surface area 547.89 m^2 ^g^−1^ was produced by using H_3_PO_4_ activating agents, and applied two-step pyrolysis. According to the American Water Work Association and based on bulk density of the investigated ACs, we recommend that most of produced ACs are suitable for treating waste water.

## 1. Introduction

Biomass has great potential as a renewable energy source, both for the richer and the developing countries [1,2]. About 140 billion metric tonnes of biomass wastes are generated every year from agriculture [3], and nearly approximately 370 million tonnes of available agricultural/biomass per year in India [4]. These wastes are one of the serious global environmental problems of world [5,6], because they accumulate in water sources, resulting in pollution such as unpleasant odours, eutrophication, high levels of biological oxygen demand and chemical oxygen demand [7,8]. Therefore, the sustainable conversion of wastes into useful products is needed [9,10]. One research trial is based on turning the biomass waste from industry and agricultural by-products as a precursor for the production of activated carbons (ACs), energy pellets and biochar production to soil amendments [11–15].

AC is a tasteless, solid, microcrystalline, non-graphitic form of black carbonaceous material with a porous structure [16]. ACs have a very porous structure with a large internal surface area ranging from 500 to 3895 m^2 ^g^−1^ [17–19], and making them versatile adsorbents with a wide range of applications. The efficiency of any AC to absorb the targeted compounds (colour, ash, colloids) depends on several factors, e.g. carbon's porosity, surface area, pore size distribution, bulk density, surface chemistry, hardness, pH, particle size and density, amount of water-soluble minerals and its total ash content [20–22]. Each of these characteristics may be of special importance depending on the projected use. Commercial production of AC depends on using raw materials, such as wood, refinery residuals, peat, coal (bituminous, subbituminous, lignite and anthracite), coke, pitches, carbon blacks and nutshells [21–24]. The ACs from agricultural by-products have the advantage of offering an effective, low cost and available replacement for the non-renewable coal-based granular activated carbons (GACs), where they have similar or better adsorption efficiency [25–28]. Owing to its high demand, high cost of production and non-renewable nature of commercial AC, this has persuaded the investigators to search for alternative materials, e.g. agriculture biomass that is cheap, renewable and sustainable.

In continuation to our work on turning of the lignocellulosic wastes as precursors for ACs and lignocellulosic composites [12–14,29,30], the present study deals with using the most available *Casuarina* fruit waste (CFW), as a precursor for production of AC. For optimizing the production route, different variables, e.g. type of chemical activating agents (H_3_PO_4_, KOH and ZnCl_2_), steps (one and two step) and temperature (600 and 700^o^C) of the pyrolysis process were examined. The performance of the produced ACs was assessed by determining the pore structure, specific surface area and surface chemistry, as well as liquid pollutants adsorption capacities.

## 2. Experimental

### 2.1. Biomass collection and analyses

CFW was collected from Kovalam Seashore, Chennai, Tamil Nadu. The proximate and biochemical properties, such as moisture, volatile matter, fixed carbon, ash, α-cellulose, hemicelluloses and lignin, were determined according to standard methods [31,32]. The elemental compositions (carbon, hydrogen, nitrogen, sulfur and oxygen (CHNS-O)) were determined by using (Perkin-Elmer 2400 Series CHNS/O Analyser) and thermo-gravimetric analysis (TGA), in the Department of Chemistry, IIT Madras.

### 2.2. Activated carbon production

ACs were prepared by one- and two-step activation methods, as described by Sugumaran *et al.* [14] and Basta *et al.* [33], but with modification in the amount of activating agent used and temperature.

#### 2.2.1. One-step pyrolysis

The air-dried biomass samples were individually treated by directly impregnating them with chemical activation agents such as phosphoric acid (H_3_PO_4_), potassium hydroxide (KOH) and zinc chloride (ZnCl_2_). The conditions of activation and pyrolysis were 10% activating agent, 4 : 1 CFW : activating agent ratio, and left overnight, then subjected to carbonization at 600°C, for 1 h. After activation, the samples were removed from the furnace and allowed to cool to room temperature. CFW only subjected to pyrolysis, without activator, was prepared as a control sample.

#### 2.2.2. Two-step pyrolysis

The biomass samples were pre-carbonized at a low temperature of 300°C for 1 h. The char was collected and impregnated with 10% of the following activators (H_3_PO_4_, KOH and ZnCl_2_), soaked also overnight. Then the samples were allowed to dry at 110°C for 5 h, followed by activation at a temperature of 700°C for 1 h. After activation, the samples were removed from the furnace and allowed to cool at room temperature.

The produced ACs were leached with 2% HCl (v/v) for 2 h and washed several times with de-ionized hot water until a neutral pH was achieved. Later, the carbon paste was dried in an electric oven at 110°C for 24 h. The AC yield was calculated by applying the formula reported in [34].

### 2.3. Characterization of activated carbons

— Physical and chemical characteristics: the pH, electrical conductivity (EC) and bulk density of the AC samples were determined as in the methods reported by Ahmedna *et al*. [35].
— Scanning electron microscope (SEM): the surface morphological changes of AC samples were investigated using an SEM (JEOL, Japan) operated at 25 kV. Oven-dried porous carbon samples were mounted on an adhesive carbon tape attached to an aluminium-stub and subsequently sputter coated with platinum for 5 min in the JFC-1100 sputter coater. The SEM magnifications were selected as ×6000.
— Brunauer–Emmett–Teller (BET) surface area: surface area measurements (m^2 ^g^−1^) of the ACs were made by low-temperature nitrogen adsorption, and applying the BET equation [36], using Micromeritics ASAP 2010 and 2020 operated at 77 K.
— Liquid adsorption studies: for liquid pollutants adsorption capacities, the iodine and methylene blue (MB) adsorption experiments were examined by the methods described in [34,37].

## 3. Results and discussion

### 3.1. Characterization of biomass wastes

The proximate, ultimate and biochemical properties of the CFW biomass sample are presented in table 1. The percentages of moisture, volatile matter, fixed carbon and ash content of CFW were 1.20%, 75.81%, 19.38% and 2.65%, respectively. The proximate results observed in this study are very close to earlier literature reports [14,38–41], which indicate the uniqueness of the biomass samples studied. Some of these chemical properties (proximate components) vary with species, nature of biomass, location within the tree and growth conditions, etc [42,43]. The *Casuarina* tree was grown in very high alkaline soil (pH < 8).

**Table 1.** Proximate, ultimate and biochemical analysis of biomass waste. (Values are mean ± s.d. of three replicates.)

	biomass waste (wt%) dry basis
parameters	*Casuarina* fruit waste (CFW)
proximate analysis	
moisture	1.20 ± 0.32
ash	2.65 ± 0.51
volatile matter	75.81 ± 0.4
fixed carbon	19.38 ± 0.21
ultimate (elemental) analysis^a^	
carbon	43.87
hydrogen	5.34
nitrogen	1.61
sulfur	0.11
oxygen	49.07
biochemical analysis	
cellulose	25.52 ± 0.01
hemicellulose	18.84 ± 0.01
lignin	46.06 ± 0.01

^a^Results based on one-time analysis.

With regard to elemental compositions (ultimate) of CFW (carbon, hydrogen, nitrogen, sulfur and oxygen), their percentages are 43.87%, 5.34%, 1.61%, 0.11% and 49.07%, respectively. This analysis revealed high contents of carbon and oxygen in the CFW, supporting the lignocellulosic structure for this waste. It is well known that the general formula of pure cellulose is (C_6_H_10_O_5_)*_n_*, it has carbon and oxygen contents of 44.4 and 49.4 wt%, respectively [44]. Whereas CFW samples contain similar contents of carbon and oxygen with 43.87 and 49.07 wt%, this is closely in agreement with data for woody biomass [44,45]. The C, H, N, O elemental analysis results of different agricultural wastes (31.80–58.30, 2.6–7.0, 0.10–6.80 and 32.05–50.20%) are very similar to earlier reports [14,15,39,40,46]. The H/C and O/C ratios are used to measure the degree of aromaticity and maturation, as is often illustrated in van Krevelen diagrams [47,48]. In this study, the ratios of H/C and O/C are 0.12 and 1.11, indicating a more graphite-like structure in the carbon. Similar results were reported by Krull *et al.* [49].

Biomass typically consists of cellulose (approx. 38–50%), a polymer of glucose, susceptible to enzymatic attack and easy to metabolize; hemi-cellulose (approx. 23–32%), primarily C5 sugar and difficult to metabolize; and lignin (approx. 15–25%), which is a complex aromatic compound, resists biochemical conversion and requires high temperatures to convert [50]. The CFW biomass contains 25.52% of cellulose, 18.84% of hemicelluloses and 46.06% of lignin. The lignin content observed in this study was higher than in previous reports, particularly in nut shells [51], as well as cellulose and hemicellulose values, which is very close to the earlier reports [40,52]. A higher lignin : cellulose ratio (biochemical) also implies lower H : C and O : C ratios (ultimately) [53].

### 3.2. Thermo-gravimetric analysis of biomass wastes

The degradation stages of TGA analysis of the selected biomass waste (CFW) is shown in table 2. It is clear that three different weight loss stages were observed in the thermal degradation of CFW. The first stage indicates the loss of entrapped water molecules (11% of weight loss, and at approx. 200°C), while, the second stage occurred at a temperature range of approximately 250–350°C, with maximum devolatilization of weight loss, and is approximately 57%. This stage ascribed the depolymerization, decarboxylation and degradation of CFW constituents (cellulose, hemicelluloses and lignin). In the third stage (approx. 350–550°C), the carbonization occurred, with 34% of weight loss. The TGA curve shows that the degradation of CFW took place at temperatures ranging from 150 to 600°C. It is well known that the pyrolysis temperature plays an important role in product distribution, yield and characteristics of AC production [14,54,55].

**Table 2.** Degradation stages of non-isothermal TGA analysis of CFW.

stage	temperature range, ^o^C	DTG peak temperature, ^o^C	mass change, %
first	RT to <100	74.6	−11
second	250–350	302.0	−57
third	350–550	445.8	−34

### 3.3. Activated carbon production and yield

Production of AC was achieved typically through two routes, physical activation and chemical activation [55,56]. Chemical activation has been successfully applied for production of AC with a better porous structure as well as surface area with narrow micropore distribution within a shorter time, when compared with physical activation [57]. The literature reported that, using H_3_PO_4_ and ZnCl_2_ as chemical agents are considered more effective, easily recovered and are less expensive activating agents for producing mesoporous carbon [58], with relatively higher yield than KOH and K_2_CO_3_ activating agents. Three factors are widely understood to influence the activation of biomass: reagent to raw material ratio, activation temperature, holding time, number of stages used (one or two stages) and chemical constituents of the precursor [29,59,60].

**Figure 1.**
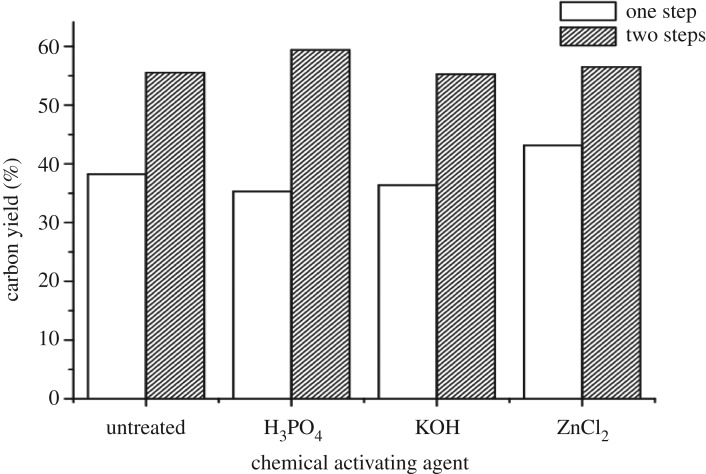
Carbon yield from CFW substrate with different activating agents and steps.

In the present study, optimizing the chemical agent and conditions for production of CFW-based AC were assessed. In this respect, three different chemical activating agents (H_3_PO_4,_ KOH and ZnCl_2_) were used, and the pyrolysis was carried out by one step (at 600°C) and two steps (at 700°C). The overall carbon yields are given in figure 1. Among the activation step, which was applied separately for different chemical agents, it is clear that the maximum carbon yield was obtained in two-step activated H_3_PO_4_ and ZnCl_2_ samples (59.39% and 56.48%, respectively). However, the lowest carbon yield was recorded in one-step activated H_3_PO_4_ (35.29%) and KOH (36.37%) treated samples. These results confirmed the explanation reported in [33]. The presence of a higher number of heteroatoms in biomass could even promote the catalytic role of activating agent (e.g. KOH) on the volatilization of the material, via breaking of C–O–C and C–C bonds, hence leading to a low carbon yield. In the two-step activation process, biomass is first carbonized, leading to a higher carbon yield than that of the previous case. KOH then reacts with carbon (not biomass, so a lower amount of KOH is required), leading to higher pore volumes and surface areas (as clear in textural properties). Excess of KOH reacts with ash, giving a soluble form which can be leached off by washing with water, hence leading to ash-free ACs, and promote the porosity. Moreover, the precursor with higher lignin content provided a higher carbon yield [15,33].

According to Basta *et al.* [13,33], the chemical constituents of precursor play a profound effect on both yield and surface area of the produced AC. Our present results indicate that, due to the lignin content (46.1%) in CFW, the high char yield is obtained, especially on applying the two steps. The percentage yield of the AC prepared depends on the different conditions *viz*. temperature, time and acid/precursor ratio [61]. Also, they are effective on the surface area of the produced AC [62].

### 3.4. Physico-chemical characteristics of activated carbons

The pH, EC, bulk density and hardness of the carbon samples were analysed and are presented in table 3. The pH values clearly reflect the activation method and chemical used [63]. The pH of the carbon directly impacts the adsorption process and affects the final pH of the adsorbent. As neutral pH is generally preferred [64], the pH of the carbon at 6–8 is acceptable for most of the application such as sugar decolourization, water treatment, etc. [65]. Some commercial carbons may have a pH of 9–10 [64]. In the present study, all the carbon samples provided alkaline pH values except H_3_PO_4_-treated CFW in both one and two activation steps. The highest pH value was recorded in the one-step activation by ZnCl_2_ (9.78) and KOH (8.72), while for two activation steps, the maximum pH value is recorded in the case of carbon produced without activating agent (8.24), followed by ZnCl_2_ AC (7.55). The pH variation in the investigated carbons always depends on the preparation, chemical activation process, presence of inorganic matter (ash components) and even washing method [23,34,65]. The EC test shows the presence of leachable ash which is considered an impurity and undesirable in AC. The EC of different AC samples has been reported to be in the range from 119.72 to 1962.26 µS cm^−1^ [12]. Table 3 shows that highest EC values were observed in ZnCl_2_ AC (1962.26 and 685.09 µS cm^−1^ of one- and two-step process, respectively), followed by H_3_PO_4_ ACs (370.13 and 280.25 µS cm^−1^, respectively) in both activation steps. A low EC value (129.54 µS cm^−1^) was recorded in untreated (without activating agent) two-step AC, followed by one-step activated KOH sample (181.08 µS cm^−1^).

**Table 3.** Physical and chemical properties of CFW AC samples. (Values are mean ± standard deviation of three replicates.)

	pH		EC (µS cm^−1^)		bulk density (g ml^−1^)		hardness (%)	
activation method/ agents	one	two	one	two	one	two	one	two
untreated	7.48 ± 0.08	8.24 ± 0.03	216.15 ± 0.42	129.54 ± 0.97	0.49 ± 0.03	0.62 ± 0.15	31.85 ± 0.15	25.00 ± 0.20
H_3_PO_4_	2.83 ± 0.03	3.78 ± 0.02	370.13 ± 0.99	280.25 ± 0.61	0.49 ± 0.02	0.67 ± 0.08	36.15 ± 0.05	29.50 ± 0.30
KOH	8.72 ± 0.02	7.33 ± 0.03	181.08 ± 1.07	261.23 ± 0.96	0.40 ± 0.06	0.61 ± 0.03	29.10 ± 0.10	24.00 ± 0.10
ZnCl_2_	9.78 ± 0.01	7.55 ± 0.04	1962.26 ± 1.06	685.09 ± 0.58	0.43 ± 0.02	0.68 ± 0.26	08.35 ± 0.05	06.55 ± 0.15

With regard to bulk density, it is an important characteristic of the carbon and invariably related to the starting material [14]. The density of the AC plays great role on adsorbate uptake. Generally, higher density carbons hold more adsorbate per unit volume [66]. In this study, all two-step AC samples provide highest bulk density (0.61–0.68 g ml^−1^), followed by one-step AC (0.40–0.49 g ml^−1^). The American Water Work Association (AWWA, 1991) has set a lower limit on bulk density at 0.25 g ml^−1^ for GACs to be of practical use. Table 3 shows that, the all investigated ACs have higher bulk density values than the AWWA; therefore, it will be suitable for water treatment purposes.

Attrition or hardness measures the mechanical strength and determines carbon's ability to withstand normal handling operations and serves as an important parameter for understanding the relative loss during the transportation, handling and regeneration [14,23,40,67]. Among one and two activations, higher hardness is observed in the case of H_3_PO_4_ and KOH (range from 29.10 to 31.85%), in the one-step process followed by two steps (range from 24.00 to 29.50%). However, in the case of ZnCl_2_-based AC samples, the lowest hardness value (6.55 and 8.35%) is observed in both the activation steps. The percentage of attrition observed in carbon, as the results indicate, depends upon the carbon density or starting materials/chemical agents of the attrition as the percentage is varied [14,23].

### 3.5. Brunauer–Emmett–Teller surface area analysis

The surface area and micropore volume of the ACs are determined by application of the BET. BET surface area describes the presence of micropore and mesopore volume of the AC materials. High surface area and porosity are very important for AC quality because it provides for the removal of large amounts of pollutants from gas or liquid streams on ACs [19,68]. The results on BET surface area (m^2 ^g^−1^), pore volume (cm^3 ^g^−1^) and average pore size in (Å) are presented in table 4. The two-step AC samples, using the three activating agents, showed higher BET values than the one-step process. Among the different activation routes, in the case of two activation steps, the maximum BET surface area is observed for H_3_PO_4_-treated carbon (547.89 m^2 ^g^−1^), followed by KOH (513.97 m^2 ^g^−1^) and ZnCl_2_ (335.08 m^2 ^g^−1^), while in the case of one-step activation, the maximum BET for H_3_PO_4_- and KOH-ACs are 262.70 and 217.55 m^2 ^g^−1^, respectively. As can be seen, one-step pyrolysis using ZnCl_2_ provides AC with a lower BET surface area (61.70 m^2 ^g^−1^) when compared with untreated carbons (carbon produced without activator). It is interesting to note that the BET of our produced AC from the two-step process is higher than in the literature [14,41].

**Table 4.** Effect of different activation agents and methods on BET surface area of AC samples^a^.

	total surface area (m^2 ^g^−1^)		pore size (Å)		pore volume (cm^3 ^g^−1^)	
activation methods/agents	one	two	one	two	one	two
untreated	146.49	323.70	766.01	1014.28	0.352	0.375
H_3_PO_4_	262.70	547.89	11 110.17	739.75	0.238	0.426
KOH	217.55	513.97	965.18	949.45	0.189	0.454
ZnCl_2_	61.70	335.08	625.69	756.84	0.739	0.418

^a^Results based on one time analysis.

The pore volume is another important parameter that characterizes the pore structure in ACs [69,70]. Table 4 shows that the one-step process provides ACs with pore volume ranging between 1.89 and 0.739 cm^3 ^g^−1^, while the two-step process provides a pore volume ranging between 0.375 and 0.454 cm^3 ^g^−1^.

### 3.6. Scanning electron microscopy

**Figure 2.**
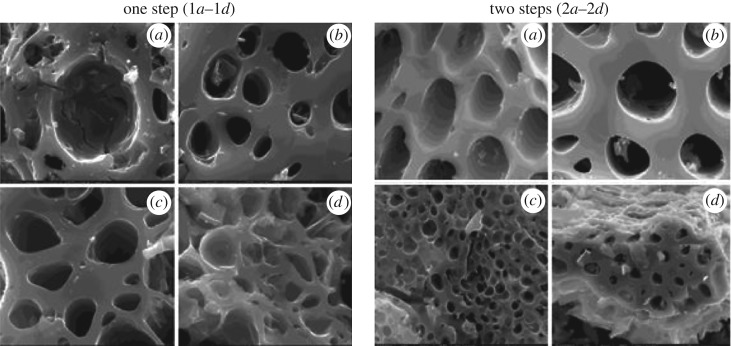
SEM micrographs of carbon samples from CFW, with magnification ×6000: (*a*) untreated carbon, (*b*) H_3_PO_4_ AC, (*c*) KOH AC, and (*d*) ZnCl_2_ AC.

SEM was carried out to find the surface morphology of different investigated carbon samples, as shown in figure 2. In the one-step activation, an SEM image of CFW untreated AC showed a large size of pore in the centre with small cracks observed at the edges of carbon samples (figure 2*a*), whereas H_3_PO_4_ treated CFW showed well-devolved oval-shaped pores and without any damage to the edges (figure 2*b*). In the case of KOH-activated CFW, the pore was irregular, oval shaped with broken edges, which looked like embeded deep holes (figure 2*c*), while AC on using ZnCl_2_, it shows irregular pores with most of the pores collapsed in the matrix (figure 2*d*).

With regard to the two-step activation, the SEM image of untreated CFW carbon showed various sizes of oval pores with smooth surface structures (figure 2*e*). H_3_PO_4_-activated CFW carbon showed shapeless pores with broken structure (figure 2*f*), while KOH AC showed oval-shaped pores with a smooth (figure 2*g*) surface. The ZnCl_2_ activated CFW carbon showed irregular pores with a broken wall in the surface (figure 2*h*). These SEM images clearly indicate that H_3_PO_4_ and KOH impregnated carbons have a more clear porous structure than the ZnCl_2_ ACs.

### 3.7. Liquid phase pollutants adsorption

**Figure 3.**
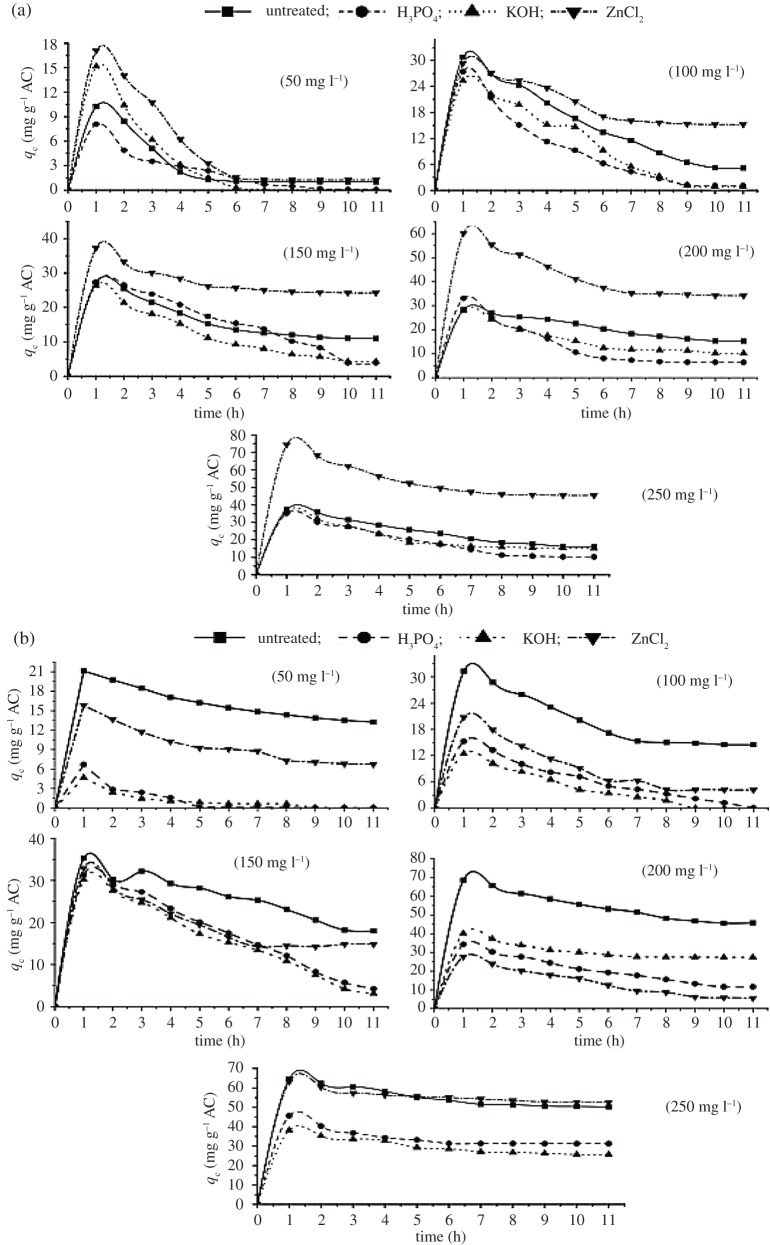
Methylene blue adsorption on CFW ACs produced by (*a*) one step and (*b*) two steps.

The results of liquid phase adsorption studies conducted using MB, iodine and molasses colour removal are presented in figure 3*a,b*. These studies were carried out to evaluate the efficiency of AC production through one-step and two-step processes to remove MB of various concentrations (50–250 mg l^−1^). The results obtained are illustrated in figure 3*a*,*b*. Among the steps of activation and type of chemical activation agent, it is clear that adsorption is higher in the case of ACs produced from two steps, using H_3_PO_4_, KOH and unactivated CFW carbon. Where, on using 250 mg l^−1^ MB concentration, the adsorption is 235.36, 229.71 and 228.67 mg g,^−1^ respectively. However, ZnCl_2_ AC samples, by both one and two steps, have low adsorption behaviour when compared with control carbon samples. This may be related to the overconcentration of ZnCl_2_, activation time, temperature, pores denaturing, etc. The KOH AC samples provide the better MB adsorption with an equilibrium time (6 h); while the other carbons registered equilibrium time at 11 h. The amount of MB adsorbed into the AC increases with increasing time, and at a specified time it levelled off, i.e. beyond which no more MB was further removed from the solution [71–73]. As can be seen, increasing the amount of carbon sample is accompanied by increasing MB adsorption.

**Figure 4.**
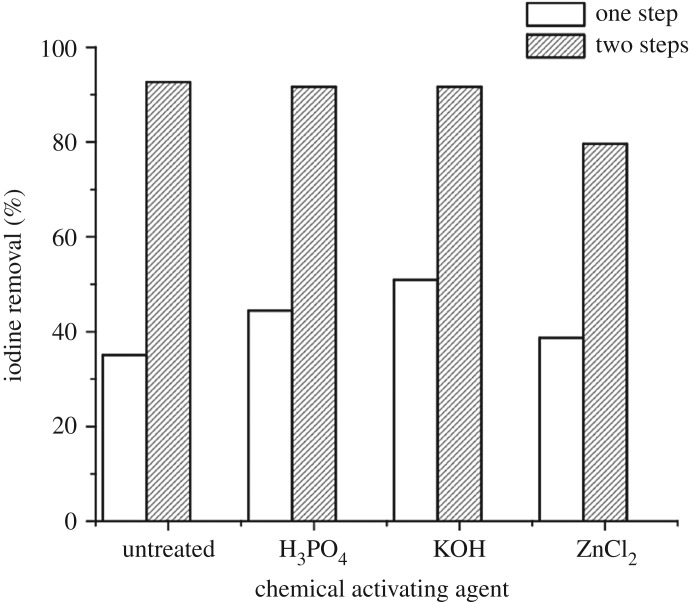
Iodine removal by different CFW AC samples.

The percentage of iodine removed by AC is also an indicator of its ability to absorb low-molecular weight compounds. The results of iodine removal by different AC samples is illustrated in figure 4. As in the trending behaviour of ACs towards MB adsorption, it can be seen that two-step ACs, using H_3_PO_4_ and KOH activating agents provided higher iodine removal (92.40% and 91.92%, respectively) than one-step carbons. Carbon from different agro-wastes was reported to remove iodine from aqueous solution in the range between 50 and 95% [34,61]. For example, the H_3_PO_4_-activated oil palm shell, oil palm fibres, oil palm empty fruit bunches, Nipa palm nut and Palmyra palm nut provide ACs with iodine adsorption values of 67.69%, 80.25%, 81.08%, 81.56% and 78.58%, respectively [12]. It is interesting to note that our CFW carbon showed higher iodine adsorption than the above-mentioned agricultural wastes. Higher efficiency of iodine adsorption has been linked to a higher surface area and the presence of largely micro and mesoporous structures [74]. It is the most fundamental parameter used to characterize the performance of AC. A high value indicates a high degree of activation and higher surface area adsorbed with higher iodine removal [75,76].

**Figure 5.**
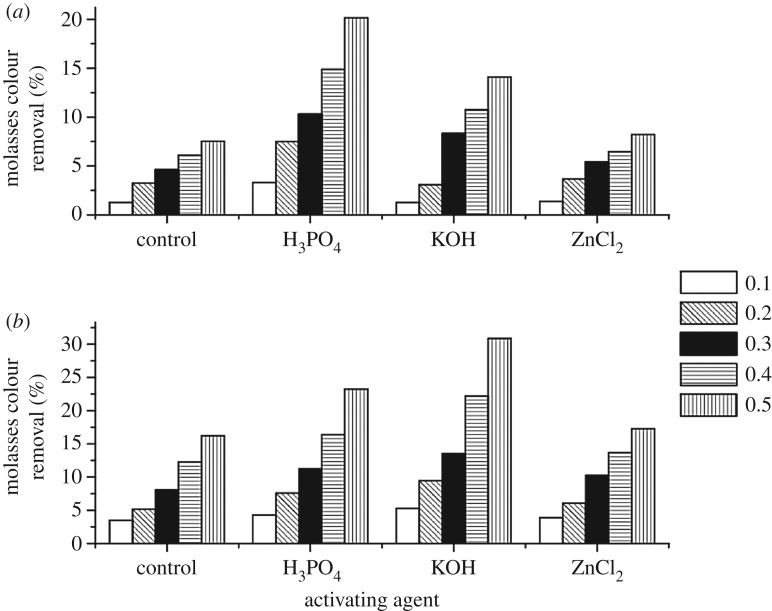
Molasses colour removal by different CFW AC samples. (*a*) One step and (*b*) two steps.

With regard to the molasses test, a varied range of molasses removal percentage is observed (1.26–30.85%), by one- and two-step AC samples (figure 5). Highest molasses colour removal is observed on using KOH (30.85%) and H_3_PO_4_ (23.24%) activating agents a for producing ACs by a two-step process, followed by one-step activation H_3_PO_4_-based carbon (20.15%). These results are closely associated with that reported earlier for sugarcane bagasse, rice husk and pecan shell, which ranged between 7.08 and 32.45% [35,41].

## 4. Conclusion

— The type of activating agent (H_3_PO_4_, KOH or ZnCl_2_) and steps of pyrolysis (one or two steps) have a profound effect on the performance of the produced AC from *Casuarina equisetifolia* fruit waste.
— The adsorption behaviour of the resulting ACs is in a good relation with their physical characterization, especially bulk density and distribution of meso to micropores.
— The carbon yield, BET surface area, removal of pollutants from liquid media (MB, iodine and molasses) was found to be higher in the case of ACs produced from H_3_PO_4_ and KOH activating agents and two-step pyrolysis, than those produced by the one-step process and using ZnCl_2_ activating agent.
— According to AWWA and based on bulk density values, the ACs produced from CFW can possibly be used as good adsorbents for various environmental applications including treatment of drinking water, and removing colour from industrial effluents.

## Data accessibility

All the data that a reader would need to attempt a replication of our work are included in the paper.
